# Ion Channels and Transporters in Muscle Cell Differentiation

**DOI:** 10.3390/ijms222413615

**Published:** 2021-12-19

**Authors:** Lingye Chen, Fatemeh Hassani Nia, Tobias Stauber

**Affiliations:** 1 Institute for Chemistry and Biochemistry, Freie Universität Berlin, 14195 Berlin, Germany; lingyechen@zedat.fu-berlin.de; 2 Zhongshan School of Medicine, Sun Yat-Sen University, Guangzhou 510080, China; 3 Institute for Molecular Medicine, MSH Medical School Hamburg, 20457 Hamburg, Germany; fatemeh.hassani-nia@medicalschool-hamburg.de

**Keywords:** bioelectricity, calcium signaling, cardiac differentiation, membrane potential, myoblast differentiation, stem cells, vascular remodeling

## Abstract

Investigations on ion channels in muscle tissues have mainly focused on physiological muscle function and related disorders, but emerging evidence supports a critical role of ion channels and transporters in developmental processes, such as controlling the myogenic commitment of stem cells. In this review, we provide an overview of ion channels and transporters that influence skeletal muscle myoblast differentiation, cardiac differentiation from pluripotent stem cells, as well as vascular smooth muscle cell differentiation. We highlight examples of model organisms or patients with mutations in ion channels. Furthermore, a potential underlying molecular mechanism involving hyperpolarization of the resting membrane potential and a series of calcium signaling is discussed.

## 1. Introduction

Vertebrates possess three types of muscle tissue classified by morphology, function, and distribution: skeletal, cardiac, and smooth muscle. The movement of living organisms and the functioning of various visceral organs rely on muscle contraction and relaxation. These processes are controlled by endogenous bioelectric signaling mediated by ion channels and transporters. The loss or dysfunction of such transport proteins usually leads to serious diseases [1,2,3,4]. For example, mutations that disrupt the voltage-gated chloride channel ClC-1 [5,6,7] and the voltage-gated sodium channel Na_v_1.4 [8,9] result in myotonia congenita and paramyotonia congenita, respectively. Ion channels and their pathogenic roles have been extensively studied in mature, excitable muscle cells [10,11,12,13]. Moreover, there is increasing evidence that ion channels also play crucial roles in muscle development. In this review, we focus on these roles of ion channels and transporters. We summarize the molecular mechanisms by which ion channels or transporters regulate the differentiation of non-excitable stem or progenitor cells during myogenesis. Additionally, we emphasize the fundamental role of endogenous bioelectrical signals in developmental processes.

Similar to muscle cells, every living cell possesses a transmembrane potential (*V*_m_) across the plasma membrane due to the uneven distribution of ions that is established or affected by various ion pumps, transporters, and channels. The range of resting membrane potentials varies between cell types. Stem cells and tumor cells tend to have a more positive, depolarized membrane potential, while terminally differentiated cells usually possess a much more negative, hyperpolarized resting potential [14,15,16,17]. For example, embryonic stem cells and skeletal muscle cells have resting membrane potentials of approximately −10 mV and −90 mV, respectively [14,16]. Ca^2+^ is one of the most important second messengers in vertebrate cells. Numerous physiological and pathophysiological processes are closely related to Ca^2+^ signaling. In general, the cytoplasmic free Ca^2+^ concentration is much lower than that of the extracellular environment. An elevated intracellular Ca^2+^ concentration can result from Ca^2+^ influx through specific Ca^2+^ channels in the plasma membrane (voltage-gated, ligand-gated, or store-operated calcium channels) or by the release of Ca^2+^ from intracellular calcium stores such as the endoplasmic reticulum, lysosomes, or mitochondria.

## 2. Ion Channels in Skeletal Myogenesis

### 2.1. Membrane Hyperpolarization

**Figure 1:**
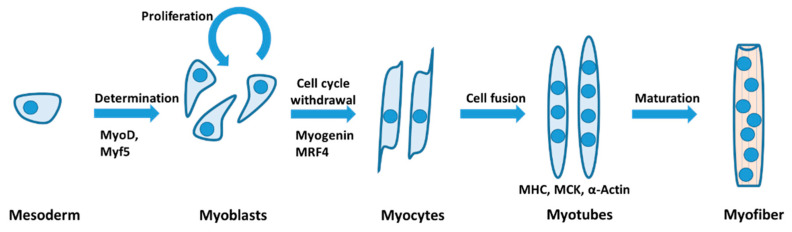
Myogenic differentiation. The scheme shows the differentiation of skeletal muscle from the mesoderm that is determined in the skeletal muscle lineage after MyoD and Myf5 expression. Myoblasts proliferate until they withdraw from the cell cycle and differentiate into myocytes, which involves myogenin and MRF4. Myocytes fuse to form myotubes that express skeletal muscle proteins myosin heavy chain (MHC), muscle creatine kinase (MCK), and α-actin. Adapted from [31].

Skeletal muscle formation occurs during the entire lifespan of vertebrates, including embryonic development, postnatal growth, and damage repair in adults [18,19]. Myogenesis from myogenic precursor cells (myoblasts) generally begins with cell cycle withdrawal, followed by the expression of muscle-specific transcription factor myogenin and the subsequent fusion of multiple cells into multinucleated myotubes (Figure 1) [20,21]. Hyperpolarization of the membrane potential is a prerequisite for skeletal muscle myoblast differentiation. This hyperpolarization can result from an efflux of cations, most likely K^+^ according to the given intracellular and extracellular concentrations, or theoretically by an influx of anions. Primary muscle progenitor cells derived from single satellite cells maintain their stem cell identity rather than undergo myogenic commitment when hyperpolarization is impaired by high external K^+^ or the Na^+^,K^+^-ATPase inhibitor ouabain [22,23,24]. More specifically, upon induction of the myogenic differentiation of human myoblasts, the activation of an ether-à-go-go (EAG) K^+^ channel has been shown to rapidly hyperpolarize myoblasts from approximately −8 mV to approximately −32 mV [25,26,27]. This is followed by a further drop in the resting membrane potential to approximately −74 mV due to the activation of the inward-rectifying K^+^ channel Kir2.1 [27,28,29]. The human EAG K^+^ current density was reported to be low in proliferating myoblasts, to increase in fusion-competent myoblasts, and to decline again in myotubes [27]. By contrast, the Kir2.1 current has been found to be expressed in 40–50% of differentiating myoblasts and in all myotubes [22,27]. Notably, the activation of plasma membrane-localized Kir2.1 channels by dephosphorylation of Tyr242 is considered one of the earliest detectable events during myoblast differentiation [28]. It occurs within the first 6 h of differentiation, several hours before the expression of the two myogenic transcription factors myogenin and myocyte enhancer factor 2 (MEF2) [22,30].

In addition to these contributors of hyperpolarization, several types of ion channels, including ether-à-go-go-related gene (ERG) K^+^ channels [29], store-operated Ca^2+^ entry (SOCE) channels [32], and volume-regulated anion channels (VRACs) [33,34], have been shown to affect the resting membrane potential of fusion-competent myoblasts. Inhibition of the human ERG K^+^ channel activity depolarized myoblasts by approximately 10 mV [29], whereas knockdown of the stromal interaction molecule 1 (STIM1) or Orai1, reducing SOCE, impaired hyperpolarization and consequently inhibited myoblast differentiation [32]. Furthermore, it has been reported that, by activating the intermediate-conductance Ca^2+^-activated K^+^ channel (IK_Ca_), extracellular 5’-guanosine-triphosphate (GTP) hyperpolarizes C2C12 cells from a mean value of −15 mV to approximately −75 mV and increases myosin heavy chain (MHC) expression [35,36,37]. VRAC is a plasma membrane channel formed by heteromers of leucine-rich repeat containing family 8 (LRRC8) members that mediates the flux of Cl^−^ and organic osmolytes in a variety of physiological processes [38,39,40,41,42]. Using an optical activity sensor [43], VRAC was shown to be transiently activated during the early stage of C2C12 (a mouse skeletal muscle myoblast cell line that expresses all five LRRC8 family members [44]) differentiation, which was also accompanied by a reduction in intracellular chloride [34]. While VRAC is not required for C2C12 proliferation [45], knockdown of the essential LRRC8A subunit [46,47] or pharmacological inhibition of its activity impaired the hyperpolarization and subsequent fusion of C2C12 myoblasts [33]. However, a VRAC-mediated efflux of Cl^−^ per se, which is evidenced by the increased cytosolic Cl^−^ upon VRAC inhibition, cannot contribute to the hyperpolarization. An explanation may be that VRAC affects other channels such as Kir2.1. VRAC was also proposed to be involved in myotube differentiation by regulating signaling independent of its ion transport activity [48].

The analysis of human patients and animal models provide in vivo evidence for the importance of ion channel function in the development of skeletal muscle. Patients with mutations in the *KCNJ2* gene, which encodes the Kir2.1 potassium channel, exhibit severe craniofacial and limb defects, such as cleft palate and brachydactyly (shortened digits) [49,50,51]. Kir2.1 knockout mice also display a cleft palate and patterning defects in their skeletal digits [52,53]. Mice with global or skeletal muscle-specific deletions of STIM1 [54,55,56] and Orai1 [57,58,59] exhibit a drastically reduced muscle mass and much smaller body size compared with their wild-type littermates. Furthermore, mice lacking the essential LRRC8A subunit of the VRAC exhibited severe growth retardation, high prenatal and postnatal lethality, and various organ abnormalities, including thin skeletal muscle bundles [60]. However, targeted deletion of LRRC8A in skeletal muscle resulted in significantly smaller myofibers without affecting total muscle mass [48].

### 2.2. Ca^2+^ Signaling

**Figure 2:**
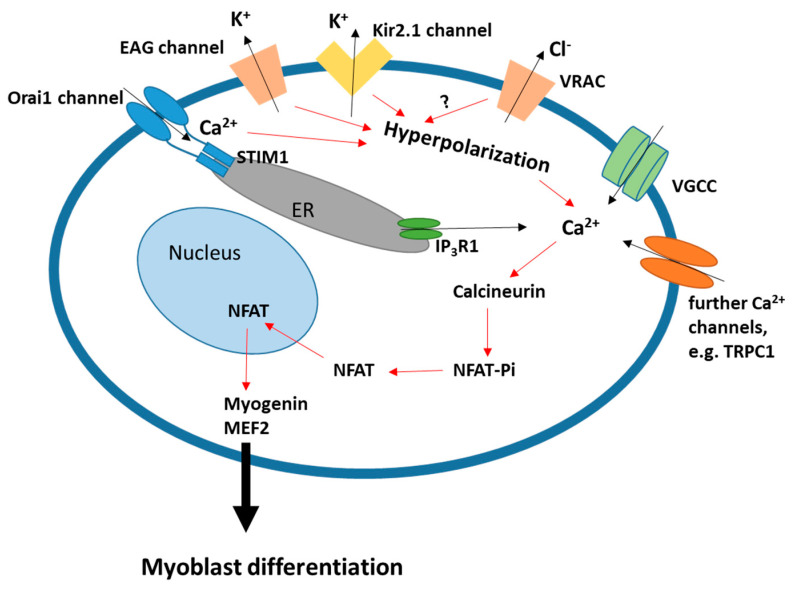
Membrane hyperpolarization and calcium signaling in myoblast differentiation. The sequential activity of EAG and Kir2.1 K^+^ channels leads to membrane hyperpolarization, which in turn is required for Ca^2+^ signaling. Ca^2+^ release from the ER, which leads to activation of SOCE, can contribute to the Ca^2+^ signal. Activity of the Cl^−^ channel VRAC contributes to hyperpolarization by an unknown mechanism. See the main text for details.

An increase in the free cytoplasmic Ca^2+^ concentration is required for the expression of myogenic transcription factors and the formation of normal-sized myotubes [23,61,62,63,64]. The hyperpolarization of human myoblasts induced by the sequential activation of EAG and Kir2.1 has been shown to trigger a small but sustained influx of Ca^2+^ through α1H T-type voltage-gated Ca^2+^ channels (VGCCs), sufficient to cause a significant increase in the resting intracellular Ca^2+^ concentration [29,62]. This cytosolic Ca^2+^ signal activates the calcineurin/NFAT pathway, thereby inducing the expression of myogenin and MEF2 (Figure 2) [23]. Another Ca^2+^-dependent pathway involving the Ca^2+^/calmodulin-dependent kinase (CaMK), is required for myogenin expression [65] but does not link to Kir2.1-induced hyperpolarization [23]. Interestingly, a 10 mV depolarization of the resting potential was observed to increase the T-type Ca^2+^ current and to raise the intracellular free Ca^2+^ concentration, thus triggering a ten-fold acceleration of human myoblast fusion [29]. However, the involvement of T-type VGCCs as a primary Ca^2+^ entry mechanism in myoblast differentiation seems to be species-dependent, as it was shown that L-type rather than T-type Ca^2+^ currents can regulate the expression of myogenin and MHC in murine C2C12 cells [66,67]. A link between L-type VGCCs and calcineurin activity has also been suggested [68].

Intracellular Ca^2+^ can also be elevated during myoblast differentiation when Ca^2+^ is released from the endoplasmic reticulum (ER) through inositol 1,4,5 tris-phosphate receptors (IP_3_Rs), followed by Ca^2+^ entry through SOCE channels [69,70,71,72,73]. Knockdown of IP_3_R1 in human myoblasts impaired both endogenous spontaneous Ca^2+^ oscillations and SOCE, which in turn reduced the activity of two key enzymes of muscle differentiation: calcineurin and CaMKII [74]. By contrast, the overexpression of IP_3_R1 not only rescued normal differentiation in IP_3_R1-silenced myoblasts but also increased the percentage of MEF2-positive nuclei after one day of differentiation [74]. In zebrafish, it was shown that, in addition to the IP_3_ receptor, the Ca^2+^-dependent ryanodine receptor (RyR) also contributes to the cytosolic Ca^2+^ signal during myogenesis upon lysosomal Ca^2+^ release by two-pore channel type 2 (TPC2) activation [75,76]. Upon ER Ca^2+^ store depletion, the Ca^2+^ sensor STIM1 triggers Ca^2+^ influx through SOCE-mediating channels located at the ER-plasma membrane junction, thereby efficiently restoring the ER Ca^2+^ content [77,78,79]. Here, two classes of channels are involved [80]: Orai channels [77,81] and transient receptor potential canonical channels (TRPCs) [82,83]. The important roles of STIM1 [32,54,55,56,84], Orai1 [32,57,58,59], TRPC1 [85,86,87,88], and TRPC4 [89,90] during myogenesis in mouse and human have been established. Silencing any of them reduced SOCE and myoblast differentiation, whereas the forced expression of STIM1 with Orai1, TRPC,1 or TRPC4 in human myoblasts increased SOCE, accelerated myoblast fusion, and produced hypertrophic myotubes [32,89]. Furthermore, the *N*-methyl-*D*-aspartate (NMDA) receptor, a subtype of ionotropic glutamate receptors, was also shown to mediate Ca^2+^ influx and to promote C2C12 myoblast fusion [91]. It is worth recalling that the graded Ca^2+^ signal involved in skeletal muscle formation depends on Ca^2+^ release from intracellular stores as well as Ca^2+^ influx from the extracellular space [29,69]. However, all of these Ca^2+^ signals are inhibited when the hyperpolarization process that increases the driving force for Ca^2+^ is blocked [23,33,36,63]. Notably, SOCE is involved in both hyperpolarization and subsequent Ca^2+^ signaling. Moreover, muscles from TRPC1 knockout mice display reduced fiber cross-sectional area and contain less myofibrillar proteins [86,92].

### 2.3. Further Molecular Mechanisms

The failure of myoblasts to exit the cell cycle leads to defective myotube formation [20,93]. It was reported that blocking the Ca^2+^- and voltage-dependent K^+^ channel KCa1.1 in human primary myoblasts increased the levels of cytosolic Ca^2+^ and activated NFκB, which resulted in enhanced cell proliferation and reduced fusion [94]. Interestingly, KCa1.1 expression in myotonic dystrophy type 1 (DM1) myoblasts was found to be significantly decreased [95], whereas introducing functional KCa1.1 α-subunits into DM1 myoblasts reduced their proliferation to normal levels and rescued the expressions of MEF2 and myogenin [94]. While constitutive overexpression of the protein called chloride intracellular channel 5 (CLIC5) partly shifted C2C12 cells from G2/M phase to G0/G1 phase, resulting in decreased cell proliferation and increased expression levels of myogenin and MHC [96], a direct effect of altered ion transport remains to be shown because it is unlikely that CLICs function indeed as chloride channels [97]. The activation of K_v_7 channels reduced proliferation and stimulated differentiation of C2C12 myoblasts [98]. In particular, it was reported that the endocannabinoid 2-arachidonoylglycerol inhibits skeletal muscle differentiation via cannabinoid type 1 receptor-mediated inhibition of K_v_7.4 channels [99]. Knockdown of K_v_7.4 reduced the expression levels of several differentiation markers, but overexpression of K_v_7.4 did not enhance myoblast differentiation [100].

Inhibition of mechanosensitive (or stretch-activated) cation channels by pharmacological blockers leads to impaired phenotypic maturation of C2C12 myoblasts, including reduced expression of sarcomeric proteins and MHC and decreased creatine kinase activity [101,102], with contradicting findings on the inhibitory effect on myogenin expression. Several further ion transport proteins have been implicated in skeletal myogenesis, including TRPC3 [103], Pannexin1 and Pannexin3 [104], connexin43 [88,105,106], two-pore domain potassium channels TASK2 and TREK1 [107], nicotinic acetylcholine receptors [63,108], transient receptor potential vanilloid 1 (TRPV1) [109,110], and Na^+^/K^+^/2Cl^−^ cotransporter 1 (NKCC1) [111]. However, the specific mechanistic roles of these proteins in myogenic differentiation have not yet been elucidated.

## 3. Ion Channels and Transporters in Cardiac Differentiation

**Figure 3:**
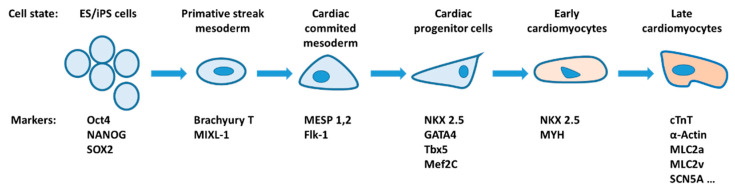
Cardiac differentiation. The scheme represents the different cell states during cardiac differentiation from pluripotent stem cells with the corresponding molecular markers. Adapted from [120].

**Figure 4:**
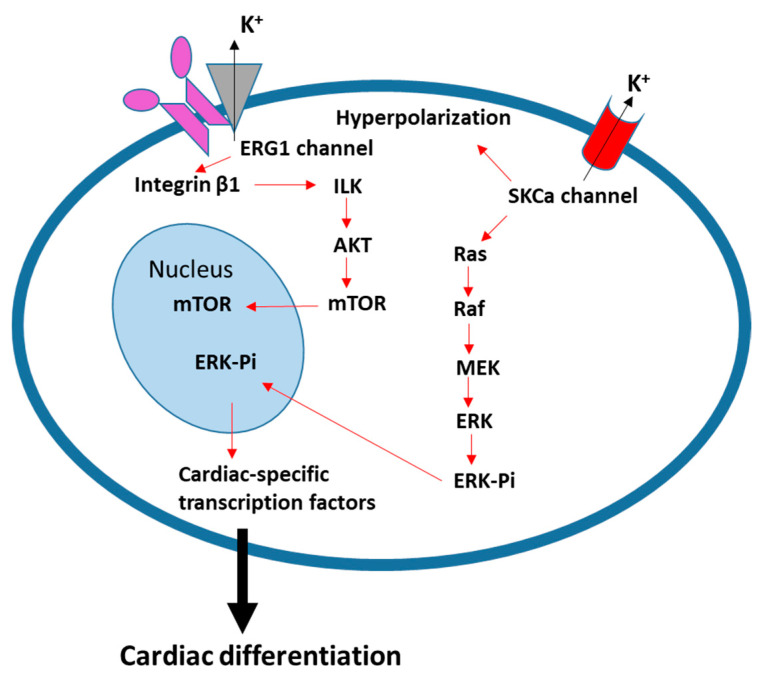
Membrane hyperpolarization and ion channel activation in cardiac differentiation. The activity of ERG1 and SKCa K^+^ channels lead to membrane hyperpolarization and signaling to cardiac differentiation. See the main text for details.

The heart is the first inner organ to form and function in the embryo. After birth, unlike skeletal muscle, the division or generation of cardiac muscle cells only occurs as a very slow process [112,113,114]. Due to the controversy or limitations surrounding cardiac progenitor cells [112,115,116], here, we focus on data describing cardiac differentiation of pluripotent stem cells (PSCs), such as embryonic stem cells (ESCs) and induced pluripotent stem cells (iPSCs). The differentiation of PSCs into cardiomyocytes is usually accomplished by the embryoid body method and is characterized by sequential expression of a series of genes: initial mesoderm and cardiomesoderm markers (brachyury T and mesoderm posterior protein 1 (MESP1)), followed by cardiac-specific transcription factors (NKX2.5, GATA4, and MEF2C), and finally cardiac-specific structural proteins (cardiac Troponin I (cTnI), MYH6, MYH7, and myosin light chain 2a (MLC2A)) (Figure 3) [112,115,117]. Interestingly, the activation of Ca^2+^-activated K^+^ channels (SKCas) (mainly the intermediate-conductance SKCa SK4) induced hyperpolarization of the membrane potential in undifferentiated murine ESCs, thereby inducing cardiac differentiation [118]. This suggests that membrane hyperpolarization precedes the signaling cascades of mesoderm commitment and cardiomyocyte specification. Notably, it was proposed that SKCa activation induces efficient cardiac differentiation of ESCs by activating the Ras-Mek1/2-ERK1/2 signal transduction pathway [118]. A recent study showed that the K^+^ channel ERG1 is involved in the cardiac differentiation of rat ESCs by interacting with integrin β1 and thus activating the AKT pathway (Figure 4) [119].

The sources of intracellular Ca^2+^ signals vary with the differentiation stage of PSC-derived cardiomyocytes and embryos maintained ex vivo [121,122,123]. In particular, the Na^+^/Ca^2+^ exchanger NCX1 is expressed during the earliest stages of heart development [123,124,125] and pharmacological blockade of NCX1 impacted on CaMKII signaling to downregulate the expression of key cardiac markers (*Nkx2.5*, *Myh6*, and *Tnnt2* (encoding cTnT)), which led to impaired differentiation and failure of cardiac crescent formation [123]. The inhibition of L-type Ca^2+^ channels that are also expressed in the early stages of cardiac development resulted in the downregulation of *Myh6* and *Tnnt2* and a reduction in beating embryoid bodies [123]. In addition, the activity of the Na^+^/H^+^ exchanger NHE1 was shown to promote cardiomyocyte differentiation; however, the mechanism has so far remained unknown [126]. In contrast with skelatal muscle differentiation, there is no clear data on a contribution of intracellular calcium stores, such as the ER, lysosomes, or mitochondria, in cardiac muscle differentiation.

A mutation that disrupts the ERG K^+^ channel activity causes severe cardiac phenotypes in human patients, including QT prolongation, functional AV conduction disturbances, and polymorphic ventricular arrhythmias [127]. Similarly, another missense mutation in the human ERG, when introduced into the orthologous mouse gene in mouse ESCs, causes developmental cardiac defects in the right ventricle and its outflow tract. Homozygous mutant offspring died in utero by embryonic day 11.5 [128]. Mutations that result in reduced voltage-dependent channel inactivation of a specific L-type Ca^2+^ channel, Ca_v_1.2, cause defects in heart development in human patients. These defects include lethal arrhythmias and congenital heart disease [129,130].

## 4. Ion Channel Activity in Smooth Muscle Cell Differentiation

Compared with skeletal muscle, there is only scarce information on the role of ion channels or transporters in vascular smooth muscle cell (VSMC) differentiation. VSMCs arise from multiple origins during embryonic development [131,132]. Although various in vitro models have been established to investigate the detailed mechanisms of deriving VSMCs from stem cells, it appears that origin-specific VSMCs possess individual regulatory mechanisms regarding the control of differentiation [133,134]. On the other hand, unlike cardiac and skeletal muscle cells, VSMCs do not terminally differentiate but maintain the ability to undergo phenotypic modulation in response to physiological and pathological stimuli, switching between a fully differentiated and contractile phenotype and a highly proliferative, migratory, and synthetic phenotype [135]. The limited progress made in defining VSMC differentiation [136,137,138] makes it more difficult to determine the role of ion channels or transporters in this process.

Many studies have presented confusing or apparently contradicting data on Ca^2+^ signaling, mostly by influx from the extracellular space [139,140,141,142,143]. Little is known about a potential role of intracellular Ca^2+^ stores in VSMC differentiation. While the lysosomal channels TRPML1 and TPC2 were shown to contribute to Ca^2+^ signaling in differentiated smooth muscle cells [144,145], a role for lysosomal as well as mitochondrial Ca^2+^ in their differentiation is unknown. The relative contributions of plasma membrane and ER Ca^2+^ channels in the plasma membrane alters when smooth muscle cells switch between a contractile and a proliferative phenotype [146,147]. However, this change in Ca^2+^ signaling does not seem to affect cell differentiation [148].

Nevertheless, it is beyond doubt that ion channels are critically involved in the development of vascular smooth muscle as well. One example is the ERG1 K^+^ channel, in which deletion results in defects in the yolk sac and intraembryonic vasculature. Treatment with the specific ERG1 antagonist dofetilide, both in vivo and in vitro, recapitulates this vascular phenotype [149]. Interestingly, a recent study demonstrated the importance of plasma membrane hyperpolarization in VSMC differentiation [150]. First, the induction of contractile differentiation of primary VSMCs by transforming growth factor TGF-β1 treatment caused hyperpolarization of the resting membrane potential. Second, TGF-β1-stimulated VSMC differentiation in the mesenchymal stem cell line C3H10T1/2 was inhibited in a dose-dependent manner in the presence of additional extracellular KCl. Furthermore, it was reported that TRPC6-mediated Ca^2+^ influx and depolarization suppressed VSMC differentiation by coordinately promoting the interaction of TRPC6 with lipid phosphatase and PTEN (phosphatase and tensin homolog) [150].

## 5. Conclusions

It is becoming increasingly clear that ion channels and transporters play conserved roles in developmental processes [151]. In contrast with transcriptional networks and signaling mechanisms, the emerging field of bioelectricity is a reservoir of new discoveries to be explored [15,16,17]. Changes in the resting membrane potential—as distinguished from transient membrane potential changes or oscillations during physiological processes—can be an instructive parameter in regulating cell fate decisions [152]. There are challenges to the investigation of the role for ion channels in cell differentiation, such as the fact that they can operate within a very narrow time window and that their regulation is not necessarily associated with changes in protein expression. The integration of various Ca^2+^ signals further adds to the complexity. Future research is required for identifying the intracellular or extracellular stimuli that control the activity of ion channels and transporters, and their downstream signaling. Elucidating the mechanisms by which ion channels and transporters promote muscle cell differentiation will lead to a better understanding of muscle development or disease and will provide insight for the development of therapeutic strategies relying on drugs or regenerative medicine.

## Abbreviations

CaMK	Ca^2+^/calmodulin-dependent kinase
CLIC5	Chloride intracellular channel 5
cTnI	Cardiac Troponin I
DM1	Myotonic dystrophy type 1
EAG	Ether-à-go-go
ER	Endoplasmic reticulum
ESC	Embryonic stem cell
GTP	5’-guanosine-triphosphate
IKCa	Intermediate-conductance Ca^2+^-activated K^+^ channel
IP3R	Inositol 1,4,5 trisphosphate receptor
iPSC	Induced pluripotent stem cell
LRRC8	Leucine-rich repeat containing family 8
MCK	Muscle creatine kinase
MEF2	Myocyte enhancer factor 2
MESP1	Mesoderm posterior protein 1
MHC	Myosin heavy chain
MLC2A	Myosin light chain 2a
Myf5	Myogenic factor 5
MyoD	Myoblast determination protein
NFAT	Nuclear factor of activated T-cell
NKCC1	Na^+^/K^+^/2Cl^−^ cotransporter 1
NMDA	N-methyl-D-aspartate
PSC	Pluripotent stem cell
PTEN	Phosphatase and tensin homolog
RyR	Ryanodine receptor
SKCa	Small and intermediate conductance Ca^2+^-activated K^+^ channel
SOCE	Store-operated Ca^2+^ entry
STIM1	Stromal interaction molecule 1
TPC2	Two-pore channel type 2
TRPC	Transient receptor potential canonical channel
TRPV1	Transient receptor potential vanilloid 1
VGCC	Voltage-gated Ca^2+^ channel
VRAC	Volume-regulated anion channel
VSMC	Vascular smooth muscle cell
